# Comparison of Postoperative Short-Term Outcomes between Tension-Free Vaginal Mesh Surgery Using the Capio™ SLIM Suture Capturing Device and Conventional TVM Surgery for Pelvic Organ Prolapse

**DOI:** 10.1155/2018/7918071

**Published:** 2018-04-01

**Authors:** Haruhiko Kanasaki, Aki Oride, Tomomi Hara, Satoru Kyo

**Affiliations:** Department of Obstetrics and Gynecology, Shimane University School of Medicine, Izumo, Shimane 693-8501, Japan

## Abstract

**Aim:**

We compared the short-term effectiveness of tension-free vaginal mesh (TVM) surgery using the Capio SLIM suture capturing device and conventional TVM surgery for treatment of pelvic organ prolapse.

**Methods:**

We retrospectively compared postoperative pain, urinary function, and length of hospital stay between 7 patients who underwent TVM surgery using the Capio device and 9 patients who underwent conventional TVM surgery.

**Results:**

There was no significant between-group difference in mean age between the Capio TVM group and the conventional TVM group (76.0 ± 5.6 years and 72.5 ± 11.5 years) or in mean operating time (86.56 ± 23.33 min and 95.28 ± 23.88 min). Four of the 7 patients in the Capio TVM group could not sense the urge to urinate after removal of the urethral catheter, but all patients in the conventional TVM group did so. The volume of the first voluntary urination was significantly smaller in the Capio TVM group than that in the conventional TVM group (102.14 ± 80.57 mL versus 472.22 ± 459.43 mL). The mean residual urine volume after the first voluntary urination was greater in the Capio TVM group than that in the conventional TVM group (285.70 ± 233.82 mL versus 34.56 ± 73.31 mL). The number of catheter days and mean maximal volume of residual urine were significantly greater in the Capio TVM group. The mean postoperative hospital stay was 6.57 ± 1.83 days in the Capio TVM group and 3.2 ± 0.42 days in the conventional TVM group. Six patients who underwent Capio TVM surgery complained of deep-seated pain in the hip region.

**Conclusion:**

Urinary function may worsen postoperatively when the Capio TVM device is used in patients with pelvic organ prolapse.

## 1. Introduction

With the increasing aging of society, 1 in 5 people are now aged 65 years or older and pelvic organ prolapse (POP) is becoming a common disease with a prevalence of approximately 30%–40% [1]. POP can seriously reduce the quality of life in women, and the lifetime risk of undergoing POP surgery is reported to be 10%–20% [2]. Although numerous surgical techniques have been described for the treatment of POP, most gynecologists in Japan have traditionally performed conventional vaginal hysterectomy, anterior and posterior colpoplasty, and circumferential suture of the levator ani muscles in women with POP, especially in those with uterine prolapse. However, the newest transvaginal technique of tension-free vaginal mesh (TVM) surgery pioneered by gynecologists in France [3] is a simpler procedure that does not require hysterectomy and is now used in many countries, including Japan. The favorable cure rate and low frequency of complications associated with TVM surgery have been extensively reported [4, 5]. Therefore, we have been using TVM surgery as a first-line surgical treatment for POP since 2009 [6].

The aim of an anterior TVM (A-TVM) repair is to suspend a cystocele by passing two arms of transobturator mesh into the paravesical region on each side. Two subvesical straps are inserted in the tendinous arch of the pelvic fascia from the anterior and posterior sides of the obturator foramen using TVM needles on both the left and right sides. The aim of a posterior TVM (P-TVM) repair is to correct a rectocele and/or uterine prolapse. In this procedure, one strap of mesh is passed into the pararectal space through the sacrospinous ligament from both sides. Therefore, to complete the conventional procedure of anterior and posterior TVM (AP-TVM) to treat POP, a total of 6 skin incisions (3 incisions on each side: 2 for A-TVM and 1 for P-TVM) are necessary for the TVM needle to access each of the ligaments.

The Capio SLIM device allows consistent placement of sutures in pelvic floor locations that are difficult to access [7]. This device enables the surgeon to catch and suture the ligament on the inner part of the pelvis in the limited surgical field available. In TVM surgery using the Capio device, arms of mesh can be driven through the sacrospinous ligament from both the anterior and posterior sites without the need for TVM needles or skin incisions. Further, this surgery can be performed with less dissection of the paravesical region than with conventional TVM surgery. Thus, in comparison with conventional surgery, TVM surgery using the Capio device is considered to be minimally invasive. We started performing TVM surgery using the Capio device for patients with POP from December 2015; however, shortly after introducing this procedure, we observed some issues. Thus, this study sought to compare the short-term effectiveness of TVM surgery using the Capio device with that of conventional TVM surgery for POP.

## 2. Methods

Seven women with POP underwent TVM surgery with the Capio device at our institution between December 2015 and April 2016. Nine women who underwent conventional TVM surgery during the same period were also selected to compare the postoperative short-term outcomes of Capio TVM surgery with those of conventional TVM surgery. All women had stage 3 prolapse according to POP-Q (Pelvic Organ Prolapse Quantification System) and underwent TVM surgery after providing written informed consent. Data were collected retrospectively from the patients' medical records. Complaints of pain and details concerning micturition were obtained from patients by direct interview. Ethical approval for this study was obtained from the ethical committee of Shimane University Hospital (approval number: 20170224-1).

The surgery was performed under general or lumbar spinal anesthesia in a lithotomy position. The conventional TVM technique has been described previously [8]. Because mesh kits for TVM surgery are not available in Japan, we used monofilament polypropylene mesh (Polyform™; Boston Scientific, Natick, MA) cut into a shape similar to that used with the Prolift system (Ethicon, Somerville, NJ) before each operation. The A-TVM procedure starts with an anterior colpotomy after local infiltration. Repair of a cystocele requires two arms of transobturator mesh to be passed on both sides in order to suspend the cystocele. On either side, both arms of the mesh are passed into the paravesical region using a modified Emmet needle. The anterior subvesical strap is inserted into the tendinous arch of the pelvic fascia. The posterior subvesical strap is inserted in the tendinous arch 1 cm from the ischial spine using a gently curved needle. In a P-TVM procedure, a posterior colpotomy is performed longitudinally and the mesh is placed under the vaginal wall. On each side, one strap of the mesh is passed into the pararectal space through the sacrospinous ligament and exteriorized via incisions located outside and below the anus.

The surgical procedure used to perform a TVM repair for POP with a Capio suture capturing device (Boston Scientific) is based on that used with the Uphold™ LITE vaginal support system. In A-TVM surgery using the Capio device, after local infiltration, a longitudinal incision is made on the anterior vaginal wall and the paravesical space is opened by blunt dissection. The mesh arms are then inserted 2 cm medial to the ischial spine on the sacrospinous ligament, which allows the mesh to be inserted through the ligament by direct fixation. This operation does not require insertion via the ligament using a TVM needle or skin incisions to insert needles percutaneously. Similarly, for a P-TVM procedure, a posterior colpotomy is performed longitudinally after local infiltration and the pararectal space is opened by blunt dissection. The mesh is placed between the vaginal wall and rectum, and the mesh arms are inserted using the Capio device through the pararectal space. This procedure allows the mesh to be inserted through the ligament without a needle or skin incisions.

One patient with anterior vaginal wall prolapse underwent A-TVM, patients with both anterior and posterior vaginal wall prolapses underwent an A-TVM and P-TVM (AP-TVM) procedure, and patients without a uterus underwent total TVM (T-TVM), in which one piece of prosthetic mesh consisting of two parts connected to each other is inserted into the anterior and posterior walls.

The operating time was defined as the time from the first incision to the completion of the last suture. The urethral catheter was not removed until the second morning after surgery. Voiding volume and residual urine volume were measured at the first voiding after removal of the urethral catheter. After the first voiding, a Nelaton catheter was passed into the bladder through the urethra, and then, the volume of urine collected was measured by using a graduated cylinder. All patients were asked if they sensed the urge to urinate before their first voiding. We measured the residual urine volume after each voiding until the residual volume became less than 100 mL (per our routine practice) and calculated the number of days on which the residual urine volume needed to be measured. We defined the second day after surgery, on which the urethral catheter was removed, as day 1. Although the patients who underwent TVM surgery were scheduled to be discharged 3 days later, the length of the postoperative hospital stay was determined on the basis of each patient's postoperative course and micturition status. The number of days to discharge was calculated from the day of surgery (defined as day 0).

Statistical analysis was performed using the unpaired Student's *t*-test. *P* values less than 0.05 were considered significant.

## 3. Results

An AP-TVM procedure was performed in 5 of the 7 patients in the Capio TVM group; one of the 2 remaining patients underwent an A-TVM surgery and the other underwent a T-TVM procedure. Six of the 9 patients in the conventional TVM group underwent an AP-TVM surgery, 2 underwent a T-TVM surgery, and 1 underwent an A-TVM procedure. The mean age was 72.1 ± 1.5 years in the Capio TVM group and 76.55 ± 5.6 years in the conventional TVM group. There was no significant difference in the mean operating time between the Capio TVM group and the conventional TVM group (95.28 ± 23.88 min versus 86.56 ± 23.33 min; Table 1).

**Figure 1:**
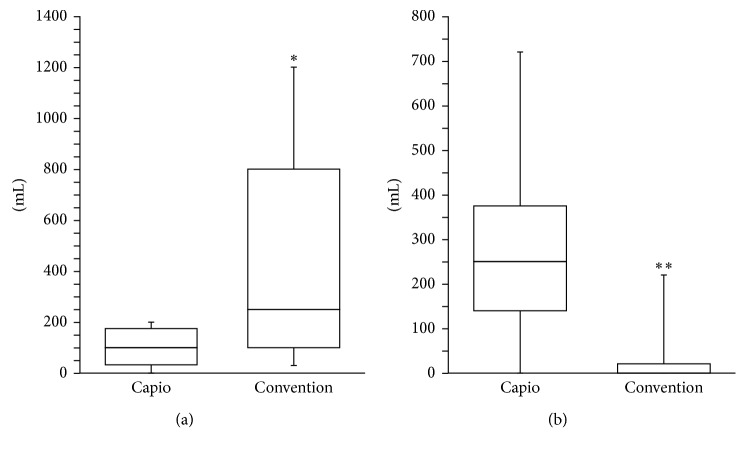
Comparison of the first voiding volume (a) and residual urine volume (b) after removal of the urethral catheter on the second postoperative day in women who underwent a TVM procedure using the Capio device or conventional TVM surgery. ∗*P* < 0.05 and ∗∗*P* < 0.01 versus the Capio TVM group. TVM, tension-free vaginal mesh.

The mean urine volume that was voided under voluntary control for the first time after removal of the urethral catheter on the second morning after surgery was significantly smaller in the Capio TVM group than in the conventional TVM group (102.14 ± 80.57 mL versus 472.22 ± 459.43 mL; *P* < 0.05, Figure 1(a)). However, the mean residual urine volume after the first voiding was significantly greater in the Capio TVM group than in the conventional TVM group (285.70 ± 2.10 mL versus 34.56 ± 73.31 mL; Figure 1(b)). Furthermore, 4 of the 7 patients in the Capio TVM group (2 AP-TVM, 1 A-TVM, and 1 T-TVM) could not sense the urge to urinate at their first urination, whereas all patients who underwent conventional TVM surgery were aware of the urge to urinate after the removal of the urethral catheter (Table 2).

The mean number of days on which the residual urine volume needed to be measured was significantly greater in the Capio TVM group than that in the conventional TVM group (3.85 ± 2.10 days versus 1.11 ± 0.32 days), as was the mean maximal volume of residual urine on these days (355.71 ± 202.80 mL versus 35.78 ± 75.50 mL). The mean postoperative hospital stay was significantly longer in the Capio TVM group than that in the conventional TVM group (6.57 ± 1.83 days versus 3.2 ± 0.42 days; Table 3).

When the patients were asked at the time of discharge from hospital about the part of the body in which they felt the most intense postoperative pain, 6 patients in the Capio TVM group complained of deep-seated pain in the hip region (including some who complained of pain in the anal canal) and 1 complained of backache. In contrast, 4 patients who underwent conventional TVM surgery felt pain at the site of the skin incisions required for posterior mesh placement using TVM needles. Three patients complained of low back pain and 2 felt deep-seated pain in the hip region (Table 4).

## 4. Discussion

Initially used in France, TVM mesh is now widely used in POP repair in many countries. A TVM procedure does not require hysterectomy and has a good cure rate with a low rate of complications [3, 4]. TVM procedures are relatively easy to learn and perform. In addition, when compared with vaginal hysterectomy, the operative procedure using TVM is considered less invasive with a shorter operating time and less intraoperative bleeding [9]. Therefore, we have performed TVM surgery as a first-line surgical treatment for POP since 2009 [6], and more than 250 cases of TVM surgery have been encountered.

We performed several TVM procedures using the Capio device because we anticipated that this new technique might be less invasive than the conventional TVM surgery. Skin incisions to guide TVM needles for ligament insertion are not necessary when performing a Capio TVM procedure. Further, avoidance of the need for TVM needles could prevent surgical complications such as injury to the bladder or rectum. Although our series is small and did not include follow-up of the long-term outcome after surgery, we did not find any merit in TVM surgery using the Capio device. Although AP-TVM takes longer time to perform than A-TVM or T-TVM surgery, the mean operating time using this device was not shorter than that required for conventional TVM surgery. In addition, the postoperative pain associated with Capio TVM surgery seems not to be less than that reported after conventional TVM surgery. Patients who underwent conventional TVM surgery complained of pain at the skin incision sites, particularly those made for posterior mesh replacement just outside and below the anus. In our Capio TVM group, there were no complaints of pain at the skin incision sites but most patients felt deep-seated pain in the hip region, which corresponded to the part of the sacrospinous ligament where the mesh arms were fixed by the Capio device. This type of pain is also observed in patients who undergo conventional TVM surgery, in which two mesh arms are passed through the sacrospinous ligament bilaterally to support the posterior mesh.

We were surprised to find that urinary function was much worse postoperatively in the patients who underwent Capio TVM surgery. After removal of the urethral catheter, more than half of the women who underwent Capio TVM surgery could not sense the urge to micturate, whereas all patients who underwent conventional surgery sensed the urge to urinate at the time of their first voiding. The volume of urine voided was decreased, and the residual urine volume was increased in the Capio TVM group. Furthermore, the requirement to measure voiding volume and the hospital stay were prolonged in this group. Similar results were reported by Rusavy et al., who showed that voiding difficulties were more frequent in patients who underwent anterior compartment repair by mesh anchored to the sacrospinous ligament using the operation kits supplied with the Capio device [10]. At present, we attribute the deterioration of voiding function in the Capio TVM group to iatrogenic injury of the nerve supply to the bladder. Postoperative voiding dysfunction is often observed in patients who undergo surgery for deep infiltrating endometriosis or radical nerve-sparing oncologic procedures [11, 12]. More aggressive bladder dissection has been reported to be associated with an increased risk of urinary retention [13]. It has also been shown that urinary retention is caused by damage to the inferior hypogastric plexus in the deep area. Our findings of markedly reduced micturition and an increased volume of residual urine in the Capio TVM group suggest that the anterior approach to the sacrospinous ligament might be the cause of the impaired innervation of voiding function.

We performed TVM surgery using the Capio device expecting that it would be less invasive in patients with POP. However, after our experience in 7 patients, we have discontinued using this device in TVM surgery. However, it remains undeniable that the more recent literature provides several articles describing the efficiency of TVM surgery using the Capio device, which typically involve less surgical dissection, shorter operating time, and low recurrence rates [7, 14]. Our relatively poor short-term surgical outcomes of this surgery might be due to the insufficient surgical technique in using the Capio device. A surgical procedure similar to this is possible and widely performed in other countries using a commercially available surgical kit, namely, the Uphold LITE vaginal support system. A surgical procedure that avoids the inconvenience of urinary tract dysfunction should be pursued when an anterior compartment prolapse repair is performed using mesh anchored to the sacrospinous ligament via an anterior approach. Further studies about the use of the Capio device are needed.

## Figures and Tables

**Table 1:** Patient and surgical details.

	Capio TVM	Conventional TVM	*P*
Patients (*n*)	7	9	
Age (years)	76 ± 5.6	72 ± 11.5	0.19
BMI	25.9 ± 4.0	24.8 ± 4.8	0.29
Procedures			
A-TVM	1	1	
AP-TVM	5	6	
T-TVM	1	2	
Operating time (min)	95.28 ± 23.88	86.56 ± 23.33	0.25

TVM, tension-free vaginal mesh; A-TVM, anterior TVM; AP-TVM, anterior and posterior TVM; T-TVM, total TVM.

**Table 2:** Urge to urinate postoperatively.

Desire to micturate	Capio TVM (*n*=7)	Conventional TVM (*n*=9)
Present	3	9
Absent	4	0

**Table 3:** Postoperative course.

	Capio TVM (*n*=7)	Conventional TVM (*n*=9)	*P*
Residual urine catheterization (days)	3.85 ± 2.10	1.11 ± 0.32	<0.01
Mean maximal residual urine volume (mL)	355.71 ± 202.8	35.78 ± 74.50	<0.01
Duration of hospital stay (days)	6.57 ± 1.83	3.2 ± 0.42	<0.01

**Table 4:** Location of most intense pain postoperatively.

	Capio TVM (*n*=7)	Conventional TVM (*n*=9)
Inner region of the hip	6	2
Skin incision for posterior mesh replacement	0	4
Low back pain	1	3

## Additional Points

*Impact Statement*. In TVM surgery using the Capio SLIM suture capturing device, the arms of the mesh can be driven through the sacrospinous ligament from both the anterior and posterior sites without the need for TVM needles or skin incisions; this surgery is thus considered to be minimally invasive. To evaluate the effectiveness of TVM surgery using the Capio device, we retrospectively compared postoperative pain, urinary function, and length of hospital stay between patients who underwent TVM surgery using the Capio device and patients who underwent conventional TVM surgery. We found that urinary function may worsen postoperatively when the Capio TVM device is used. Further studies investigating the use of the Capio device are needed.
